# *Enterococcus faecalis* induces H₂O₂-mediated epithelial cell death and enhances *Candida albicans* virulence in oropharyngeal candidiasis

**DOI:** 10.1128/msphere.00822-25

**Published:** 2025-12-31

**Authors:** Roberto Vazquez-Munoz, Amit Ranjan, Martinna Bertolini, Angela Thompson, Pegah Mosharaf Ghahfarokhy, Alannah Harnden, Clarissa J. Nobile, Takanori Sobue, Paola Vera-Licona, Anna Dongari-Bagtzoglou

**Affiliations:** 1 Department of General Dentistry, University of Connecticut 365941, Farmington, Connecticut, USA; 2 Department of Periodontics and Preventive Dentistry, University of Pittsburgh 6614, Pittsburgh, Pennsylvania, USA; 3 Department of Molecular and Cell Biology, University of California Merced 33244, Merced, California, USA; 4 Health Sciences Research Institute, University of California 33244, Merced, California, USA; 5 Department of Cell Biology, University of Connecticut 242843, Farmington, Connecticut, USA; Kyungpook National University, Daegu, Republic of Korea

**Keywords:** *Candida albicans*, *Enterococcus*, fungal-bacterial interactions

## Abstract

In immunosuppressed humans with oropharyngeal candidiasis (OPC) and in mice with experimental OPC, *Candida albicans* infection is associated with a bacterial imbalance characterized by significantly reduced oral microbiome diversity and the expansion of enterococcal and streptococcal species, which may exacerbate oral mucosal pathology. In this study, we applied an unbiased genome-wide transcriptomic profiling approach to shed further mechanistic light on the role of indigenous enterococcal communities in mucosal infection in a mouse model of cancer chemotherapy-associated OPC. Transcriptomic profiling of tongue tissues revealed a wide-ranging, barrier-compromising molecular activity of resident enterococci that explains the previously observed attenuation of fungal mucosal invasion with antibiotic treatment in this mouse model. Mechanistically, we validated the pathogenic potential of resident bacteria by showing that enterococci isolated from mice with OPC produce hydrogen peroxide (H_2_O_2_) and induce oral epithelial cell death through apoptosis and necrosis *in vitro*. We also discovered that *C. albicans* increased enterococcal H_2_O_2_ production. These findings uncover a novel mechanism of pathogenic synergy between *C. albicans* and *Enterococcus faecalis,* which may be responsible for increased epithelial barrier damage and mucosal invasion by *C. albicans* hyphae during cancer chemotherapy.

**IMPORTANCE:**

Chemotherapy-induced mucosal barrier injury and immune suppression increase susceptibility to oropharyngeal candidiasis (OPC), a debilitating fungal infection. Our study uncovers a previously unknown pathogenic interaction between *Candida albicans* and *Enterococcus faecalis*, by showing that indigenous enterococci produce H_2_O_2_, which contributes to oral epithelial cell death during fungal infection. By integrating transcriptomics with functional assays, we demonstrate that enterococci compromise epithelial integrity independently of fungal burdens, highlighting the role of the bacterial microbiota in driving tissue damage. These findings emphasize the need to consider bacterial-fungal interactions in managing OPC and suggest that targeting the microbial crosstalk could be a promising adjunctive strategy in immunocompromised hosts.

## INTRODUCTION

*Candida albicans* colonizes the alimentary tract of 30%–70% healthy individuals within the first few weeks of life and persists without causing disease (1). This microorganism becomes a pathobiont responsible for most oral mucosal fungal infections, especially in immunocompromised individuals (2). One well-recognized risk factor for oropharyngeal candidiasis (OPC) is cytotoxic cancer chemotherapy due to its myelosuppressive effect combined with direct injury to the mucosal barrier (3). 5-Fluorouracil (5-FU) is a cancer chemotherapy drug associated with increased risk for OPC (4, 5). In addition to the increased frequency of oral candidiasis, an oral bacterial dysbiotic shift, with a reduction in salivary bacterial alpha diversity, has been identified in these patients (4).

It is possible that, in myelosuppressed cancer patients undergoing chemotherapy, resident or transient oral bacterial species that escape immune clearance form synergistic relationships with *C. albicans*. In support of this hypothesis, using a mouse model that recapitulates immune and mucosal barrier consequences of cytotoxic chemotherapy in humans (6), we showed that OPC is associated with almost complete loss of mucosal bacterial diversity. Importantly, in this model, oral *Candida* biomass increased in parallel with the biomass of indigenous bacterial species, with over 90% being *Enterococcus faecalis* (7). We also reported that enterococcal clearance with antibiotics ameliorated invasive oral candidiasis in these mice, without affecting fungal burdens (7).

Despite these advances in knowledge, the mechanistic role of resident bacteria in OPC pathogenesis remains poorly defined. Here, we applied an unbiased genome-wide transcriptomic profiling approach to shed further mechanistic light on the role of indigenous enterococcal communities in mucosal pathology in a mouse model of chemotherapy-associated OPC (6, 7). Based on validated transcriptomic data, we provide evidence of wide-ranging, barrier-compromising molecular activities of resident enterococci that would explain the previously observed attenuation of fungal mucosal invasion with antibiotic treatment in this mouse model (7). We functionally validated the role of resident bacteria by showing that enterococci isolated from mice with OPC produce hydrogen peroxide and induce oral epithelial cell apoptosis and necrosis *in vitro*. Importantly, we discovered a novel mechanism of bacterial-fungal pathogenic interaction since *C. albicans* promotes enterococcal H_2_O_2_ production. Our findings point to a novel mechanism of cooperative virulence between *C. albicans* and *E. faecalis,* which may be responsible for increased damage of the epithelial barrier and mucosal invasion by *C. albicans* during cancer chemotherapy.

## MATERIALS AND METHODS

### Microorganisms and culture conditions

*C. albicans* strain SC5314 (ATCC MYA-2876) was sub-cultured in Yeast Extract (Sigma, USA)-Peptone (Gibco, USA)-Dextrose (J.T.Baker, US) (YPD) broth and incubated aerobically at 30°C in an orbital shaker, overnight. A catalase homozygous deletion mutant (orf19.6229, *cat*1 Δ/Δ) was constructed in the isogenic strain background SN250, derived from SC5314, using a markerless CRISPR/Cas9 system optimized for use in *C. albicans*, and vetted using PCR diagnostics, as previously described (8). In brief, a 20 bp guide RNA (gRNA) was designed to target the locus of interest and direct Cas9 to generate a site-specific double-strand break. Donor DNA (dDNA) with at least 50 bp of homology to the *C. albicans* genome flanking the target region was provided to facilitate homologous recombination and repair. Transformants were plated on YPD supplemented with 200 µg/mL nourseothricin to select for colonies containing integrated Cas9 and gRNA constructs. Correct genome modifications were verified by colony PCR. Successfully edited strains were then counter-selected by plating on SD medium lacking leucine to promote loss of the CRISPR/Cas9 plasmids and the nourseothricin resistance cassette. Colonies that grew on SD–leu media and were sensitive to nourseothricin were confirmed to have lost the selection markers. Two independently constructed deletion mutants (strains X and Y) using different gRNA constructs, with a verified negative catalase test, were used to confirm the experimental results and rule out off-target effects. Verified strains were stored at –80°C for long-term preservation. All primers used for deletion and PCR validation of orf19.6229 are listed in Table S1. Prior to experiments, *C. albicans* strains were washed in PBS (×2) and adjusted to final concentrations using a Neubauer chamber.

*E. faecalis* strain #13 (Ef13) was isolated from the tongue of a C57BL/6 female mouse (Jackson Laboratories, USA) with 5-FU-associated OPC (7, 9). Ef13 was subcultured overnight in BHI broth (BD Difco), anaerobically at 37°C, and was grown in fresh BHI broth to OD_600_ = 0.8–1, at 37°C under anaerobic conditions prior to use in all experiments.

### Murine 5-FU chemotherapy model of OPC

**Fig 1:**
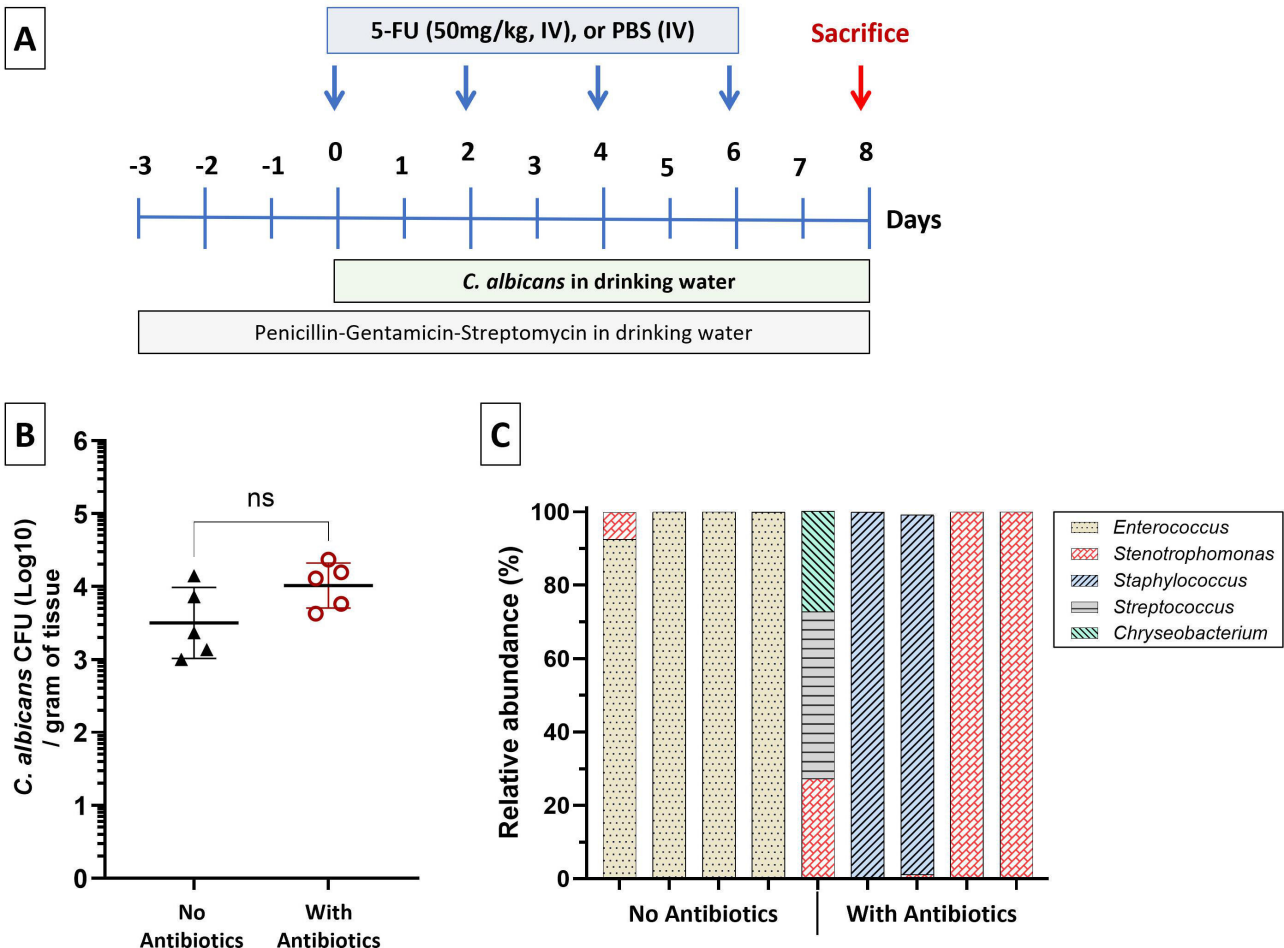
Effect of triple antibiotic regimen on *C. albicans* burdens and indigenous cultivable bacteria. (**A**) Schematic of the OPC mouse model: mice received 5-FU (50 mg/kg IV) every 48 h, with continuous *C. albicans* exposure via drinking water. Antibiotics (penicillin, streptomycin, gentamicin) were administered in drinking water as indicated. Mice were sacrificed on day 8 for tissue collection. (**B**) Tongue fungal burdens in mice with or without antibiotic treatment. Fungal burdens are expressed as mean ± SD log CFU/g of tissue. Data shown are from five mice/group. The antibiotic treatment had no significant impact on fungal burdens (unpaired Welch’s t-test). (**C**) Genus-level identification of cultivable oral bacteria in mice with OPC. Colonies recovered from tongues were identified by 16S rRNA V4 sequencing in four mice without antibiotic treatment and five mice that received antibiotics during fungal infection. As shown here, in mice without antibiotics, almost all bacterial OTUs aligned with the genus *Enterococcus*, whereas no enterococci were recovered from mice receiving antibiotics.

We used a mouse model of intravenous chemotherapy that reproduces mucosal and bone marrow changes in cancer patients receiving 5-FU (6). In this model, *C. albicans* SC5314 causes highly invasive candidiasis in the tongue submucosal tissues within 6–8 days of chemotherapy (7). Briefly, after a week of acclimatization, 4-to-6-week-old female C57BL/6 mice (Jackson Labs) received 50 mg/kg 5-FU (Sigma), intravenously (IV, via lateral tail vein) every 48 h for 6 days. Control groups received PBS. Mice were inoculated with an overnight *C. albicans* suspension culture added daily in the drinking water (6 × 10^6^ yeast/mL of water). To prevent the rise of indigenous enterococci in *Candida*-infected mice, we used a combination of three antibiotics (penicillin 1.5 mg/mL, streptomycin 2 mg/mL, and gentamicin 0.1 mg/mL) in the water, starting 3 days prior to fungal inoculation and continuing throughout the experimental period (Fig. 1A). Eight days after fungal inoculation, mice were sacrificed, and tongues were processed for microbial culture, immunohistochemistry, or mammalian cell RNA isolation. To estimate fungal burdens, tongues were homogenized using a tissue homogenizer, homogenates were diluted (1:10), and 100 µL aliquots were plated on Sabouraud Dextrose Agar containing chloramphenicol (10 µg/mL, Sigma). In some experiments, homogenates were also plated on CHROMagar medium (DRG International) to rule out the overgrowth of indigenous *Candida* species. A minimum of five mice per group was used in each experiment. Mouse experiments were performed in compliance with the federal regulations described in the Animal Welfare Act, the recommendations in the Guide for the Care and Use of Laboratory Animals of the National Institutes of Health, and the guidelines of the University of Connecticut Institutional Animal Use and Care Committee (IACUC). This study was approved by the IACUC committee of UCONN Health, protocol #AP-201567.

### Analysis of oral cultivable bacteria in *Candida*-infected mice

To evaluate the effect of the triple antibiotic regimen on oral bacteria in *Candida*-infected mice, tongue homogenates were plated on BHI Agar supplemented with Nystatin (250 U/mL, BHYN). Bacterial cultures were incubated aerobically for up to 5 days. Distinct colony morphotypes from each mouse tongue were identified at the genus level by 16S sequencing of the V4 region, amplified using 515F and 806R primers with Illumina adapters and bar codes on the 3′ end, as described previously (7). Sequences were clustered using a 97% similarity cutoff and classified using Mothur’s version of the Ribosomal Database Project classifier (Mothur 1.39.5) (10). The relative abundance of each genus was expressed as the percentage of each bacterial operational taxonomic unit over the total bacterial reads in each sample. All 16S V4 DNA sequencing raw data have been deposited in the NCBI SRA database under accession number PRJNA531284.

### RNA isolation, sequencing, and analyses

To evaluate the effect of antibiotics on the oral mucosal transcriptome in *Candida*-infected mice, three tongue tissues from each of the two infected groups (with and without antibiotics) were harvested and stored in RNAlater at −80°C until processing. Samples were thawed on ice for 15 min, and RNA isolation was carried out using the RNeasy Kit (Qiagen) with a few modifications. Briefly, thawed samples were homogenized in 800 µL of RLT buffer containing 1% of β-mercaptoethanol (Biorad) using a tissue homogenizer. Subsequently, 800 µL of phenol:chloroform:isoamyl-alcohol (Invitrogen) was added to the homogenate in 2 mL screw-capped tubes. Following centrifugation, the aqueous phase was collected, and an equal volume of 70% molecular-grade ethanol was added. Kit instructions were followed for subsequent column separation steps. DNase treatment was performed using the TURBO DNA-free kit (Invitrogen). RNA was analyzed for quality using the Experion automated gel electrophoretic system (Biorad), and only samples with RNA integrity values >7 were used for RNA sequencing.

cDNA libraries were prepared using the TruSeq RNA kit (Illumina) and subjected to poly(A) enrichment according to the kit protocol. cDNA fragments (100 nucleotides from each end) were sequenced using the Illumina NextSeq platform. The raw reads obtained from each sample were combined and subjected to quality check using FASTQC (https://www.bioinformatics.babraham.ac.uk/projects/fastqc/). Sickle was used to trim the reads, which were again evaluated using FASTQC (https://github.com/najoshi/sickle). Reads were aligned using HISAT2 (Hierarchical Indexing for Spliced Alignment of Transcripts) against the *Mus musculus* (GRCm38) index (11). The aligned reads were then converted into a binary format using SAMtools, and the duplicates were removed using the tool PICARD (12). The counts were calculated using the HTseq-count program, and comparisons were done between the antibiotics and non-antibiotic OPC groups (13). False discovery rate (FDR) ≤ 0.01 and adjusted *P*-values ≤ 0.05 were applied to identify significantly differentially expressed (DE) genes using the DESeq2 package. Samples within a group were compared using principal component analysis and a sample-to-sample distance matrix. Finally, the R software was used to compare the two groups, and DE genes (DEGs) were tabulated and subjected to further downstream analysis. GeneXplain (https://genexplain.com/) and Ingenuity Pathway Analysis (IPA), utilizing the “Core Analysis” function (Ingenuity Systems Inc.), were used for gene ontology (GO) enrichment analysis (14, 15). Cytoscape was used to visualize the networks, and standard R scripts were used to generate heat maps. Custom scripts were deposited at the GitHub repository (https://github.com/VeraLiconaResearchGroup/oropharyngeal-candidiasis).

### Validation of DEGs in tongue tissues

Validation of a subset of DEGs was performed by RT-qPCR, using an aliquot of the RNA samples subjected to RNA-seq. A total of 1µg of RNA/sample was converted into cDNA using the SuperScript III First-strand Synthesis (Invitrogen) kit. Quantitative PCR was performed with SYBR Green and the CFX96 real-time instrument (Biorad). Expression levels of selected genes were compared to those of *Gapdh*, which was used as an internal control. Target genes consisted of *Serpina3n*, *Runx1*, *Capn1*, *Vegfa, Il6st, Bcl2*, and *Xbp1*. The primers used for these genes are listed in Table S1.

Protein expression levels of Serpina3n, with the highest mRNA differential expression, were further validated by immunofluorescence staining of paraformaldehyde-fixed, paraffin-embedded tongue tissues. Briefly, deparaffinized sections were permeabilized using 0.2% Triton X-100, followed by antigen retrieval for 10 min at 94°C. After blocking in 10% normal goat serum, slides were stained with hamster anti-mouse Serpina3n monoclonal antibody (5 µg/mL, Millipore, MABC1182) followed by Goat anti-hamster IgG conjugated with Alexa Fluor 488 (1:1,000, Invitrogen, A21110). A secondary antibody only was used as a negative control. Nuclear staining (blue) was performed with Hoechst dye (1:5,000 in PBS). The mean fluorescence intensity of the Serpina3n signal was quantified with Image J in 3 mice/group, in at least six microscopic fields/group.

### Evaluation of apoptosis in tongue tissues

Mucosal cell apoptosis was evaluated using the DeadEnd Colorimetric TUNEL System, according to the manufacturer’s instructions (Promega, Madison, WI). To confirm results with this assay, tissues were stained for active caspase-3, using a rabbit polyclonal antibody recognizing mouse cleaved caspase-3 (1:100, Cell Signaling Technology, antibody# 9661), followed by tissue processing with the rabbit-specific HRP/DAB (ABC) Detection IHC Kit (Abcam, ab64261).

### Epithelial cell cytotoxicity assays

To test the ability of *E. faecalis* and *C. albicans* to induce oral epithelial cell cytotoxicity, we performed *in vitro* assays using the RealTime-Glo Annexin V Apoptosis & Necrosis kit (Promega), according to the manufacturer’s instructions. Briefly, human oral keratinocytes (OKF6-TERT2 immortalized cells, kindly provided by Dr. Rheinwald) were seeded at 10^4^ cells per well in poly-D-Lysine-coated white opaque 96-well plates (Corning). Cells were infected 48 h later with *E. faecalis* (strain Ef13, at 10^5^ or 10^6^ cells/well), *C. albicans* (strains SC5314, SN250, or *cat1*Δ/Δ, at 10^4^ yeasts/well), or both *E. faecalis* and *C. albicans* (at 10^5^ and 10^4^ cells/well, respectively), with/without catalase (40 Units/well); medium-only and no-cell wells were included for controls and background correction. Luminescence (apoptosis) and fluorescence (necrosis) signals were measured at 0, 24, and 48 h on a Synergy II plate reader (BioTek). Positive controls included 20 µM H_2_O_2_ (apoptosis) and 0.1% Triton X-100 (necrosis). Cytotoxicity assays were performed in at least three independent experiments with technical triplicates.

In a different experimental design, *C. albicans* SC5314 (5 × 10^4^ yeast cells/well), *E. faecalis* strain Ef13 (5 × 10^6^ cells/well), or a combination of both were added on the surface of transwell inserts (0.4 µm pore size, Corning) in six-well plates, to physically separate them from epithelial cells. Microorganisms were allowed to interact with epithelial cells in this manner for 5 h, and proportions of live, pre-apoptotic, and apoptotic cells were measured using a flow cytometer (MACSQuant Analyzer 10). Negative controls included media only, and positive controls included media supplemented with 20 µM of H_2_O_2_. At the end of the epithelial-microbial interaction period, epithelial cells were collected using the Accutase Cell Detachment Solution (EMD, Millipore) and stained simultaneously with 5 μL FITC-conjugated annexin-V and 5 μL propidium iodide (PI) for 20 min in the dark (BD Biosciences). Pre-apoptotic cells are positive for annexin-V and negative for PI, whereas late apoptotic cells are positive for both annexin-V and PI.

### *C. albicans* metabolic activity assay

To assess the effect of *E. faecalis* on the metabolic activity of *C. albicans*, yeast cells of strain SN250 were suspended in phenol-free RPMI media supplemented with 10% BHI and 10% FBS, seeded at 10^4^ cells/well in 96-well plates, and allowed to adhere and germinate for 3 h. *E. faecalis* strain Ef13 was subsequently added at 10^5^ cells/well with *C. albicans* and incubated in a CO_2_ incubator for 2 h. To assess the role of H_2_O_2_ in these interactions, both catalase homozygous deletion mutants (*cat1*Δ/Δ(X) and *cat1*Δ/Δ(Y)), generated in the SN250 background, were tested to rule out off-target effects. To rescue the mutant phenotype, catalase (40 Units/well) was added to some wells. At the end of the coculture period, 100 µL/well of XTT solution containing coenzyme Q0 (0.25 mg/mL XTT and 40 µg/mL coenzyme Q0, Sigma) was added to each well, and plates were incubated at 37°C and 5% CO_2_ for 2 h. Supernatants were transferred into new plates, and optical densities were measured at 450 nm, with a 630 nm reference filter. Fungal metabolic activity was expressed as %fungal viability  = *x*/*y* *100, where *x* is the OD_450_ of experimental wells (*C. albicans* with *E. faecalis*), and *y* is the OD_450_ of control wells (*C. albicans* only).

### Hydrogen peroxide assay

The release of H_2_O_2_ by *E. faecalis* in culture supernatants was measured using the Amplex Red hydrogen peroxide/peroxidase assay kit (Invitrogen). Briefly, overnight anaerobic cultures of strain Ef13 were diluted to an OD_600_ of 0.1 and subcultured anaerobically in BHI to an OD_600_ of 0.8. Cultures were resuspended in BHI and added to a 96-well plate, at 10^6^ cells/well. To test the effect of *C. albicans* on H_2_O_2_ production, overnight cultures of *C. albicans* (strains SC5314, SN250, or *cat1*Δ/Δ(X)) in YPD were resuspended in BHI and added to *E. faecalis* at 1:10 or 1:100 fungal:bacterial cell ratio. Controls included bacteria and fungi alone and non-viable 4% paraformaldehyde-fixed organisms. Colorimetric changes were measured at OD_590_ in 30-min intervals over the course of 3 h at room temperature. Results are expressed as H_2_O_2_ concentrations calculated using a standard curve according to the manufacturer’s instructions. Production of H_2_O_2_ was confirmed by inoculating strain Ef13 on MRS agar plates supplemented with horseradish peroxidase (10 mg/mL) and TMB (250 mg/mL).

## RESULTS

### Changes in mucosal gene expression associated with antibiotic depletion of indigenous enterococci in *Candida*-infected mice

To explore the role of indigenous enterococci in modulating the mucosal responses in OPC, we compared host gene expression in mice infected with *Candida albicans* that had either intact or depleted indigenous bacteria due to antibiotic treatment (Fig. 1A). As expected, triple antibiotic treatment did not significantly affect fungal burdens (Fig. 1B), but caused complete depletion of cultivable enterococci, which represented 93%–100% of oral cultivable bacteria in *Candida*-infected mice (Fig. 1C). In the antibiotics group, five other genera (*Lactobacillus*, *Staphylococcus*, *Stenotrophomonas*, *Pseudomonas,* and *Chryseobacterium*) were detected across samples composing about 25%–100% of the cultivable bacteria in each mouse tongue. The residual cultivable bacterial composition differed among the five mice in the antibiotics group (Fig. 1C).

To explore how enterococcal depletion influenced epithelial gene expression, we performed comparative transcriptomic analyses between the two *Candida*-infected groups. Initial transcriptomic analyses were carried out with three samples in each of the two OPC groups (antibiotics, no antibiotics). In the six sequencing libraries, more than 99% of sequences were aligned with the mouse reference genome GCRm39. Based on the variability of replicates within the two groups as assessed by principal component and distance matrix analyses, one sample from the antibiotics group was excluded from further analyses.

**Fig 2:**
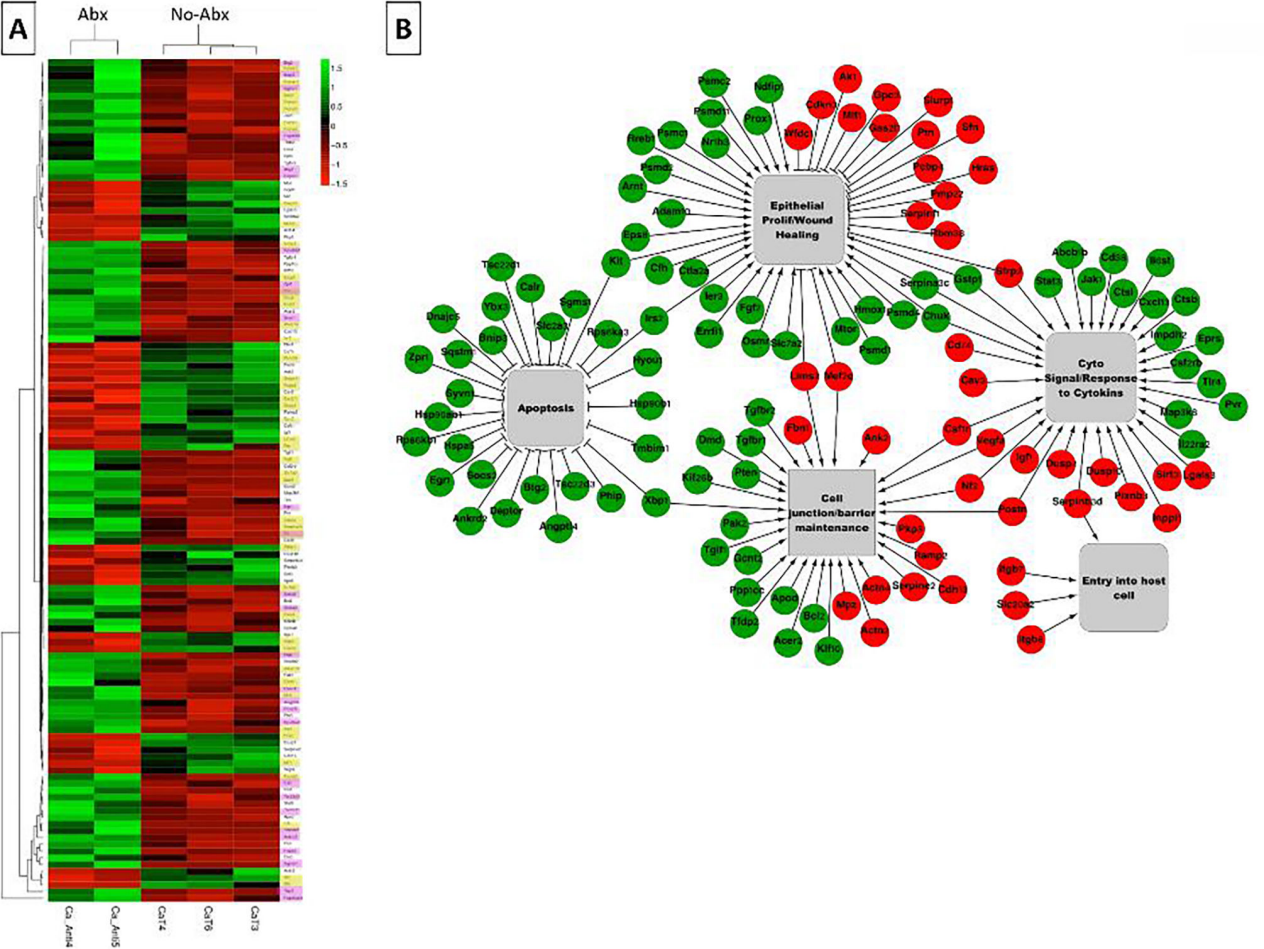
Antibiotic-driven depletion of enterococci reprograms epithelial gene expression linked to apoptosis, repair, cytokine signaling, and barrier function. (**A**) Heatmap of DEGs in tongues from *Candida*-infected mice with (Abx) or without (No-Abx) treatment. Columns represent individual mice; rows represent the 125 DEGs meeting |fold-change| ≥ 2 and FDR ≤ 0.01 and *P* ≤ 0.05. Heatmap depicting z-scores of the 125 DEGs that were identified to be significantly overrepresented in the following GO terms: Apoptosis (genes marked in pink), epithelial proliferation/wound healing (genes marked in yellow), cell junction/barrier maintenance, cytokine signaling, and entry to host cells. (**B**) Functional enrichment network of DEGs. Nodes represent genes (circles) or enriched biological processes (gray boxes); edges denote curated relationships. The network is rendered in cytoscape based on GeneXplain/IPA analyses. Network visualization of DEGs (and their effect) is shown across five functional categories related to the following GO terms: Epithelial proliferation/wound healing, cytokine signaling/response to cytokines, cell junction/barrier maintenance, apoptosis, and entry into host cells. These five biological processes consisted of a total of 125 DEGs. Nodes in green are overexpressed, and nodes in red are underexpressed genes in antibiotics compared to no antibiotics. Edges between genes and GO terms with arrows represent a positive effect, while blunt arrows represent a negative effect. Please see Fig. S2 for the component GO terms contributing to each functional category. Notice that *C. albicans* infection in mice receiving antibiotics shows the upregulation of apoptosis inhibitors and of genes with a positive effect on epithelial proliferation, while epithelial proliferation inhibitors are downregulated. These results are consistent with bacterial aggravation of *C. albicans* mucosal tissue damage.

The MA plot of normalized transcript counts in the *Candida*-infected antibiotic-treated group in comparison to the group not treated with antibiotics highlighted the presence of broad transcriptional reprogramming following microbial depletion (Fig. 1 supplemental). In the two groups, a total of 1,037 genes were differentially expressed, comprising 488 genes that had higher expression and 549 genes that had lower expression in the mice treated with antibiotics. We defined significantly DEGs as those with a minimum twofold change in expression, a *P*-value of <0.05, and a FDR < 0.01. We identified 198 genes with significant differential expression in the antibiotics group. Of these, 81 genes had higher expression while 117 genes had lower expression in the antibiotics group, compared to the group not receiving antibiotics (Fig. 2A and Supplemental data). These findings indicate that indigenous enterococci profoundly shape the transcriptional landscape of the oral mucosa during *Candida* infection.

### Functional enrichment analysis of bacterially influenced genes

To understand the biological significance of the gene expression changes associated with enterococcal depletion, we performed functional enrichment analysis of the 198 significantly DEGs using the GeneXplain software. Most of the DEGs (85 of the overexpressed and 43 of the genes with lower expression in the antibiotics-treated mice) were clustered in five major biological processes relevant to mucosal integrity (Fig. 2B and Supplemental data). These included apoptosis (*n* = 28 genes), epithelial cell proliferation and wound healing (*n* = 59 genes), cytokine signaling/cytokine response (*n* = 35 genes), cell junction/barrier maintenance (*n* = 31 genes), and entry into host cells (*n* = 4 genes). Twenty-nine genes were functionally linked to more than one category, reflecting overlap in barrier, cell death, and immune regulatory functions. For example, *xbp1,* which inhibits apoptosis, also plays a role in cell junction/barrier function. The individual biological processes that were combined to form the five larger functional groups are shown in Fig. S2. Thus, enrichment analyses revealed that most DEGs were functionally related to epithelial biological processes that maintain the integrity of the mucosal barrier. Importantly, DEGs negatively influencing epithelial apoptosis and positively influencing proliferation were significantly enriched in mice that received antibiotics. Additionally, antibiotic treatment induced suppression of genes that inhibit wound healing. DEGs related to cytokine responses were also abundant, supporting the role of bacteria in the mucosal immune response to *Candida* infection (Fig. 2B and Fig. S1). These findings suggest that enterococci disrupt epithelial homeostasis during OPC by driving apoptosis, suppressing wound repair, and impairing barrier function.

### Validation of differentially expressed host genes

**Fig 3:**
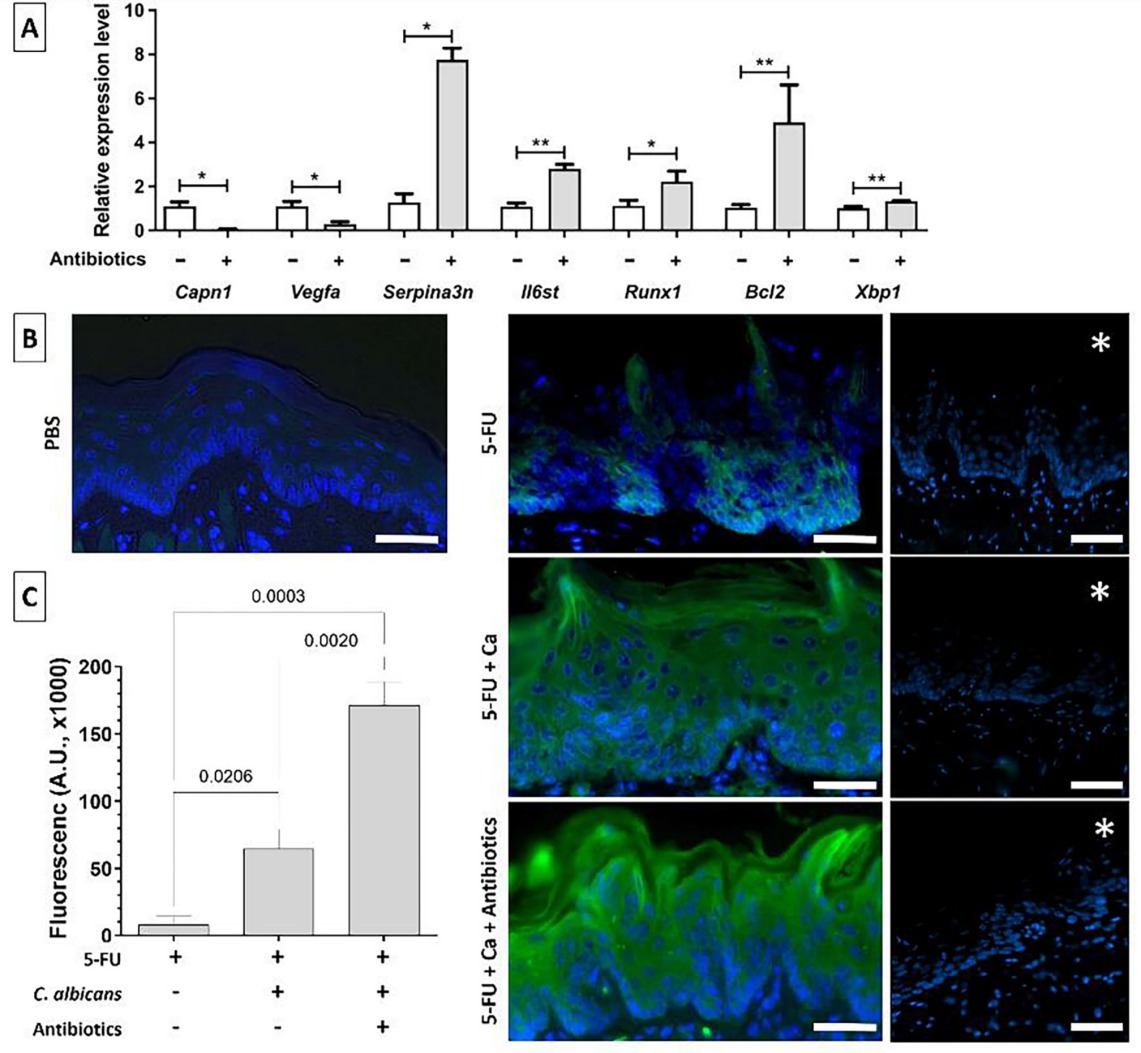
Validation of differential gene expression. (**A**) RT-qPCR confirmation of seven DEGs in the antibiotics/no antibiotics mouse groups. Data shown are fold changes relative to a housekeeping gene (*gapdh*) using the Δ/Δ_CT_ method. *Serpina3n*, associated with mucosal barrier protection, was significantly upregulated in the antibiotics group, whereas *calpain1* (*capn1*) associated with barrier breach was significantly downregulated. Other genes (*il6st*, *runx1*, *bcl2*, *xbp1,* and *vegfa*) were validated as well. Bars show mean ± SD in three tongues/group, *P < 0.01, **P < 0.05. (**B**) Immunofluorescence staining of tongue tissue sections showing increased Serpina3n protein (green signal) in the antibiotics group. Cell nuclei are counterstained with Hoechst (blue). Representative tissue section stains are shown from the control (PBS), 5-FU-treated, 5-FU-treated-*Candida*-infected (5-FU + Ca), and antibiotics (5-FU + Ca + antibiotics) groups. Asterisks (*) on images denote sections processed without primary antibody (negative control staining). Bars = 50 µm. (**C**) Quantification of green fluorescence intensity in the three 5FU-treated experimental groups using Image J. Bars represent mean fluorescence arbitrary units (A.U. ×1,000) ± SD, in three mice/group. Significantly higher (*P* = 0.002) serpina3n protein expression in the antibiotics compared to the no antibiotics OPC groups (unpaired Welch’s corrected t-test).

To validate transcriptomic findings and confirm biologically meaningful changes in host responses, we selected a subset of DEGs for RT-qPCR analysis. These genes were chosen based on their roles in apoptosis, cytokine signaling, and epithelial barrier regulation and included calpain 1 (*capn1*), serine peptidase inhibitor, clade A, member 3N (*serpina3n*), vascular endothelial growth factor A (*vegfa*), runt-related transcription factor 1 (*runx1*), interleukin 6 signal transducer (*il6st*), B-cell leukemia/lymphoma 2 (*bcl2*), and X-box binding protein 1 (*xbp1*) (Fig. 3A). Consistent with the RNAseq data, *capn1* and *vegfa* had significantly lower expression, while *il6st*, *runx1*, *bcl2*, and *xbp1* had significantly higher expression in the antibiotics-treated group. These changes support the conclusion that enterococcal depletion reduces apoptotic signaling and promotes epithelial survival and regeneration.

We further focused our differential expression analyses on epithelial barrier function-associated genes, since *C. albicans* invades deep into the muscular tissue of the tongue in this model (7). Among these, three variants of the mouse *Serpina3* gene (n, k, and c) encoding Serpina3 were significantly enriched in the antibiotics-treated group based on IPA gene enrichment analysis (14-fold enrichment, *P* < 2.3 × 10^−3^). These variants were all underexpressed at the transcriptional level in mice that were not treated with antibiotics. RT-qPCR confirmed higher *serpina3n* gene transcription (Fig. 3A) and immunofluorescence staining revealed a stronger Serpina3 signal in the epithelial cell layer of *Candida*-infected mice treated with antibiotics, confirming increased Serpina3n protein expression in these mice (Fig. 3B and C). Together, these results validate the transcriptomic data and highlight *serpina3n* as a key epithelial defense factor suppressed by enterococci during OPC. The concurrent downregulation of *capn1*, a protease that can facilitate barrier breach (16), further supports the hypothesis that enterococci actively impair epithelial integrity.

### Experimental validation of the role of indigenous enterococci in mucosal cell death

**Fig 4:**
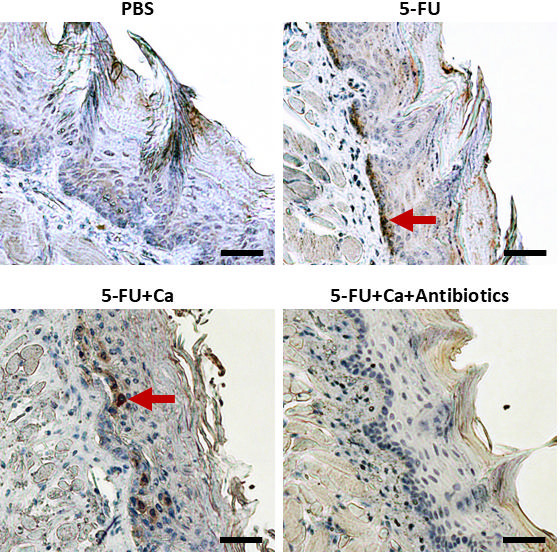
Epithelial apoptosis is reduced in antibiotic-treated mice with OPC. Immunohistochemical staining for cleaved caspase-3 using a rabbit polyclonal antibody. Positively staining apoptotic cells are shown in brown (red arrows); nuclei are counterstained with hematoxylin. Representative tissue section stains are shown from the control (PBS), 5-FU-treated, 5-FU-treated-*Candida*-infected (5-FU+Ca), and antibiotics (5-FU+Ca + antibiotics) groups. Bars = 50 µm.

**Fig 5:**
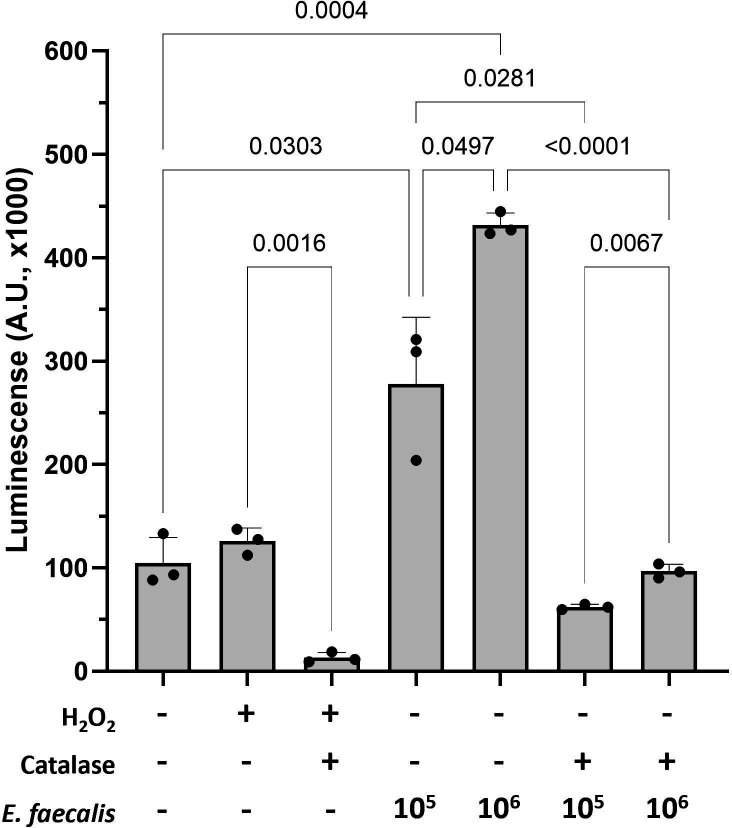
*E. faecalis* induces dose-dependent epithelial apoptosis mediated by H_2_O_2_. OKF6/TERT-2 cells were infected with increasing cell numbers (10^5^ or 10^6^ cells/well) of *E. faecalis* strain Ef13. Exogenous H_2_O_2_ (20 µM) was used as a positive control, and catalase (40 U/well) was added to test the role of this oxidant in apoptosis. Apoptosis of OKF6-TERT2 keratinocytes after 24-h infection was quantified using the RealTime-Glo Annexin V luminescence assay. Results are expressed as mean arbitrary luminescence units (A.U. ×1,000 ± SD, Brown-Forsythe uncorrected one-way ANOVA). Results shown are from a representative of three independent experiments with three technical replicates.

A large number (*n* = 28) of genes functionally related to apoptosis inhibition were more highly expressed in *C. albicans*-infected mice that received antibiotics. This suggested that epithelial cell apoptosis may be more prominent in *C. albicans*-infected mice, where enterococci are the dominant bacteria. To explore this further, we investigated apoptosis levels in the tongue tissues of mice using TUNEL staining, as well as immunohistochemistry staining for active caspase-3. Using both methods, we found that mice with OPC that received antibiotics had lower apoptosis staining, raising the possibility that enterococci induced epithelial cell apoptosis (Fig. 4 and Fig. S3). To experimentally test this hypothesis, we evaluated the ability of the mouse *E. faecalis* strain Ef13 to induce apoptosis when cocultured with OKF6-TERT2 oral epithelial cells. A significant increase in apoptosis was observed with increasing amounts of enterococci (Fig. 5), confirming their role in causing oral epithelial damage.

Hydrogen peroxide is produced by most oral lactic acid bacteria, and oxidative stress caused by H_2_O_2_ induces apoptosis, leading to cell death (17–19). Strain Ef13 oxidized TMB when exposed to air for 15 min after anaerobic growth, which indicated that this strain produces H_2_O_2_ (20) (Fig. S4). To investigate the role of enterococcal H_2_O_2_ in epithelial apoptosis, we added catalase in epithelial-enterococcal cocultures, which completely inhibited epithelial cell apoptosis (Fig. 5). These findings supported the conclusion that *E. faecalis* can directly induce oral epithelial cell apoptosis and that enterococcal H_2_O_2_ is a key mediator in this process.

### *C. albicans* increases enterococcal H_2_O_2_ production and is protected from oxidative damage by expressing catalase

**Fig 6:**
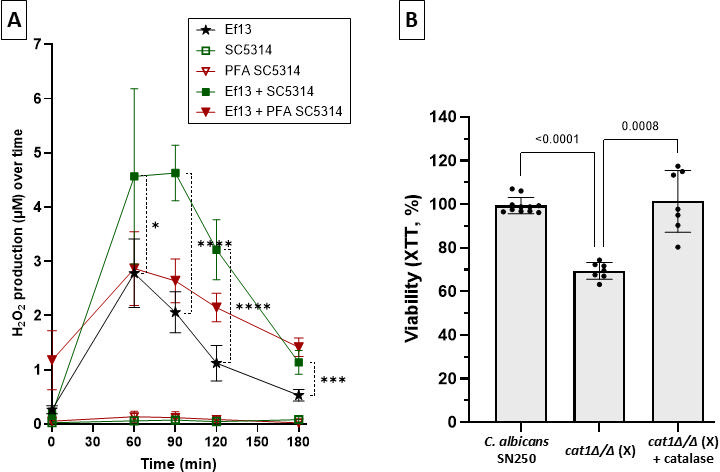
*C. albicans* enhances enterococcal H₂O₂ release, and catalase limits fungal oxidative damage. (**A**) Kinetics of extracellular H₂O₂ release during aerobic culture of *E. faecalis*, *C. albicans,* and their combination in BHI broth media. H_2_O_2_ in culture supernatants was measured with the Amplex Red hydrogen peroxide/peroxidase assay kit. *E. faecalis* alone (strain Ef13), *C. albicans* alone (strain SC5314), and paraformaldehyde-fixed *C. albicans* were used as controls. Results represent means ± SD from three independent experiments. Live *C. albicans* significantly increased H_2_O_2_ from *E. faecalis* after 60 and 90 min of interaction. ***P < 0.001, ****P < 0.0001 (uncorrected two-way ANOVA with Fisher’s LSD test). (**B**) Effect of *C. albicans* catalase on oxidative damage from *E. faecalis. C. albicans* strain SN250 or an isogenic catalase homozygous deletion mutant (*cat1*Δ/Δ strain(X)) was allowed to interact with *E. faecalis* for 2 h at 1:10 fungal:bacterial ratio, and fungal metabolic activity was measured by the XTT assay. Catalase (40 U/well) was added to rescue the mutant phenotype. Fungal metabolic activity was expressed as %fungal viability  = *x*/*y* *100, where *x* is the OD_450_ of *C. albicans* with *E. faecalis,* and *y* is the OD_450_ of *C. albicans* only. The metabolic activity of the catalase mutant is significantly compromised by *E. faecalis*, while the metabolic activity of the reference strain is not significantly affected. Adding catalase rescues the mutant from oxidative damage. Results represent means ± SD from three independent experiments with technical triplicates. *P* values shown are from Brown-Forsythe uncorrected one-way ANOVA (Welch’s t test correction).

Under continuous aerobic growth conditions on TMB-supplemented MRS agar strain, Ef13 did not produce a pigment, suggesting that H_2_O_2_ production is affected by environmental stimuli (Fig. S4). To test whether *C. albicans* affects bacterial H_2_O_2_ production, we cocultured the two organisms aerobically and measured the release of this oxidant over time (Fig. 6A). *C. albicans* in monocultures did not produce detectable extracellular H_2_O_2_. When incubated alone, strain Ef13 released H_2_O_2_ after 60 min, which dropped gradually over the 3-h observation period. When co-cultured with live *C. albicans,* H_2_O_2_ release was significantly higher at 60 min of interaction and increased further at 90 min, before gradually diminishing. At all observation time points, H_2_O_2_ production by Ef13 was significantly higher when it was cocultured with *C. albicans* (Fig. 6A). To further test whether metabolically active *C. albicans* cells were required to induce significant H_2_O_2_ release by strain Ef13, we exposed Ef13 to fungal cells killed by 4% paraformaldehyde. While non-viable fungi did not significantly increase H_2_O_2_ after 60–90 min of interaction, we observed a small but statistically significant increase in H_2_O_2_ after 2–3 h, suggesting that both metabolic and non-metabolic interactions between the two organisms are likely involved in this process (Fig. 6A).

Because enterococci may generate fungicidal levels of H_2_O_2_, we questioned whether *C. albicans* metabolic activity can be negatively influenced by strain Ef13 and whether this was H_2_O_2_-dependent. The XTT assay showed that the metabolic activity of the *C. albicans* reference strain SN250 was not affected by coculture with strain Ef13 but was significantly reduced in both isogenic catalase mutants. Furthermore, adding catalase rescued both mutants from metabolic toxicity, confirming that catalase protects fungal cells from *E. faecalis*-derived H_2_O_2_ (Fig. 6B and Fig. S5).

### *Candida*-enterococcal interactions increase oral epithelial cell death *in vitro*

**Fig 7:**
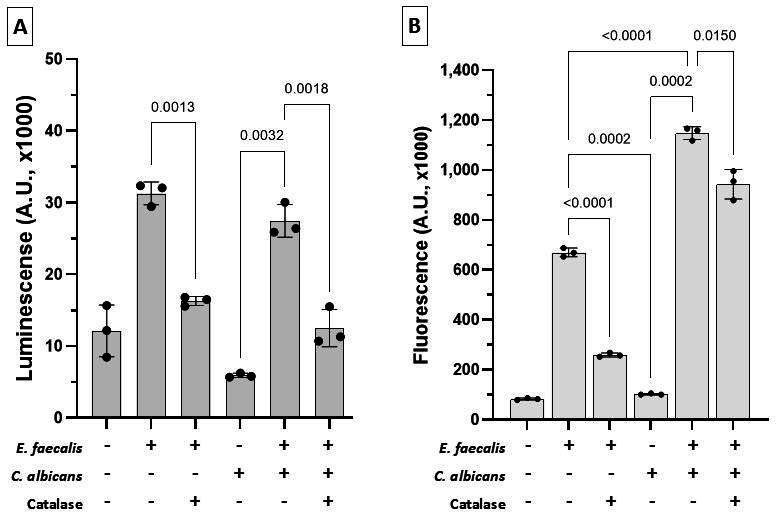
Co-infection with *E. faecalis* and *C. albicans* increases oral epithelial cell cytotoxicity. (**A**) Apoptosis: OKF6-TERT2 keratinocytes were exposed for 24 h to *C. albicans* SC5314, alone or together with Ef13 (1:10 fungal-bacteria cell ratio), with/without catalase (40 U/well). Apoptosis was quantified by luminescence using the RealTime-Glo Annexin V assay. Bars show mean luminescence A.U. ×1,000 ± SD in a representative of three independent experiments with technical triplicates. Dual-species infection did not increase apoptosis over the Ef13 mono-infection. Apoptosis in single- and dual-species infection was completely inhibited by catalase, confirming the role of H_2_O_2_ in this process. (**B**) Necrosis. Necrosis was quantified in the same cultures after 48 h of infection by measuring fluorescence. Bars show fluorescence A.U. ×1,000 ± SD in a representative of three independent experiments, with technical triplicates. Ef13 alone significantly increased necrosis, and catalase attenuated this effect (*P* < 0.0001). Dual infection induced greater necrosis than either organism alone (*P* = 0.0001 vs Ef13; *P* < 0.0002 vs *C. albicans*), and catalase partially rescued the epithelial cells from necrosis, showing that H_2_O_2_ is partially responsible for necrosis. All *P* values are from Brown-Forsythe uncorrected one-way ANOVA (Welch’s t test correction).

Since we found increased amounts of H_2_O_2_ in co-cultures of *C. albicans* and *E. faecalis,* we hypothesized that the two organisms may synergize to aggravate epithelial oxidative cell damage. To begin to explore this possibility, we exposed epithelial cells to *E. faecalis* Ef13, *C. albicans* SC5314, or both organisms together, and measured apoptosis using two different assays. In the 96-well luminescence assay, infection of OKF6-TERT2 cells with both organisms did not increase the H_2_O_2_-dependent apoptosis above the levels induced by *E. faecalis* alone (Fig. 7A). Consistent with the luminescence assay, the flow cytometry assay showed that exposure to both organisms together did not increase apoptosis compared to *E. faecalis* alone (Fig. S6). Our results suggest that, under these epithelial co-culture conditions, *C. albicans* does not increase enterococcal H_2_O_2_ levels sufficiently to further augment epithelial apoptosis caused by *E. faecalis*.

In OKF6-TERT2 cells, *E. faecalis* alone induced significant epithelial cell necrosis after 48 h of infection, which was almost completely inhibited by the addition of catalase (Fig. 7B). This suggests that most enterococcal-induced epithelial cell death is a consequence of H_2_O_2_-mediated apoptosis. In cells co-infected with both *E. faecalis* and *C. albicans,* there was a significant increase in cell death above the effect of each organism alone, which was partially inhibited by catalase (Fig. 7B). Overall, these experiments support the role of resident enterococci in increasing mucosal cell damage caused by H_2_O_2_-induced oxidative stress. Our findings also suggest a cooperative relationship between the two organisms in augmenting epithelial cell death that is independent of H_2_O_2_-induced oxidative damage.

**Fig 8:**
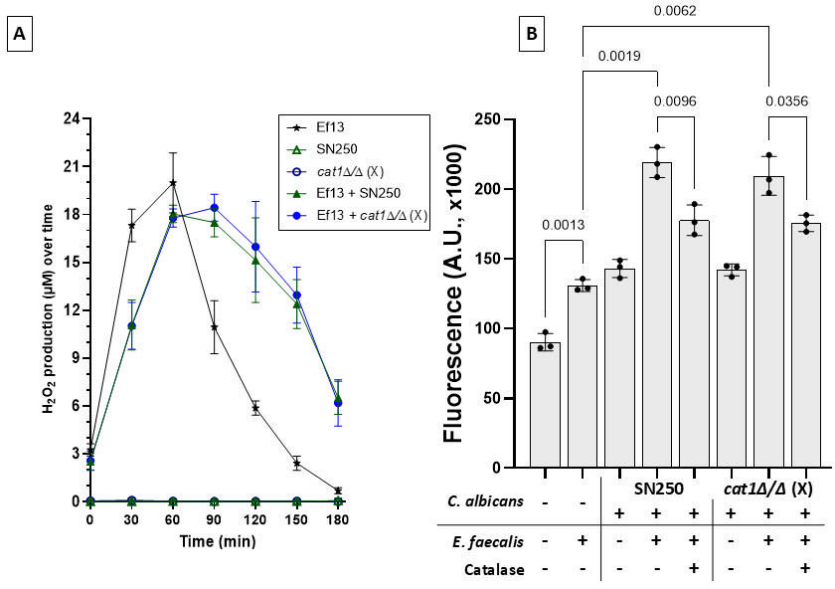
Fungal catalase does not protect oral epithelial cells from oxidative damage. (**A**) Kinetics of extracellular H₂O₂ release during aerobic culture of *C. albicans* reference strain SN250, *cat1*Δ/Δ(X) isogenic mutant, and their combination with *E. faecalis* strain Ef13 (1:10 fungal:bacterial ratio) in BHI broth media. H_2_O_2_ in culture supernatants was measured with the Amplex Red hydrogen peroxide/peroxidase assay kit. Results represent mean ± SD from three independent experiments. Both *C. albicans* reference strain SN250 and the catalase mutant significantly increased H_2_O_2_ by *E. faecalis* after 90 min of interaction, with no difference between the two strains. (**B**) Epithelial cell necrosis. OKF6-TERT2 keratinocytes were exposed for 48 h to *C. albicans* reference strain SN250, *cat1*Δ/Δ(X) isogenic mutant, and their combination with *E. faecalis* strain Ef13 (1:10 fungal:bacterial cell ratio) with/without catalase (40 U/well). Bars show fluorescence A.U. ×1,000 mean ± SD in a representative of three independent experiments, with technical triplicates. There is no difference between the reference and mutant strains in causing epithelial necrosis alone or in combination with Ef13. Catalase partially inhibited cell necrosis in fungal-bacterial co-infected cells. *P* values are from Brown-Forsythe uncorrected one-way ANOVA (Welch’s t test correction).

Although *C. albicans* catalase is primarily thought to be an intracellular enzyme, some experimental evidence suggests that it may also be cell wall-associated, or even secreted extracellularly (21, 22). We thus questioned whether fungal catalase could play a role in curtailing the epithelial damage inflicted by H_₂_O_₂_ during fungal-bacterial co-infection. To address this question, we first compared the levels of enterococcal H_2_O_2_ stimulated by the reference and *cat1*Δ/Δ strains (Fig. 8A). We reasoned that if fungal catalase acts extracellularly, then higher amounts of H_2_O_2_ would be detected in the *E. faecalis*-catalase mutant co-culture supernatants, compared to the reference strain. However, we found no significant difference in the amounts of H_2_O_2_ detected in co-culture supernatants between the reference strain SN250 and the catalase mutant (Fig. 8A), arguing against this possibility. In agreement with this finding, there was no significant difference between the reference and the *cat1*Δ/Δ strains in epithelial cell death caused by fungal-bacterial co-infection (Fig. 8B). Together, these results show that fungal catalase does not play a role in epithelial cell protection from oxidative damage.

## DISCUSSION

We identified a novel mechanism of bacterial-fungal synergy in OPC, in which the *Candida*-induced bacterial dysbiosis, characterized by an expansion in resident enterococci, promotes epithelial cell apoptosis and necrosis. *E. faecalis* is a ubiquitous colonizer of mucosal sites, and like *C. albicans,* it is an opportunistic pathogen in susceptible hosts (23). *Candida* and enterococci are often co-isolated from clinical samples (24), and in immunosuppressed mice with OPC, both *C. albicans* and *E. faecalis* disseminate hematogenously to the liver (25), suggesting they cooperate to breach the oral mucosal barrier. In healthy adults, oral enterococci are generally considered transient commensals, and carriage rates are below 10%. However, the oral carriage rate of *Enterococcus* species (predominantly *E. faecalis*) in patients with underlying systemic diseases such as cancer rises to 60% (26, 27). These populations are also high risk for the development of OPC (28). In the 5-FU chemotherapy murine model of OPC, *E. faecalis* is the most dominant oral bacterial species, representing up to 99% of the total bacterial microbiota (7). Therefore, this model offers a unique opportunity to study the role of *E. faecalis* in fungal pathogenesis.

We previously showed that *Enterococcus* depletion with antibiotic treatment in the 5-FU OPC model significantly reduced *C. albicans* oral mucosal invasion (7). We thus hypothesized that the overgrowth of these species activates host response pathways that facilitate mucosal barrier breach by fungi. To further explore the role of *E. faecalis* in this process, we compared the transcriptome of 5-FU-treated mice, where invasive candidiasis is associated with *E. faecalis*-dominated dysbiosis, and 5-FU-treated mice receiving antibiotics, which have a more superficial infection (7). Transcriptomic profiling revealed that depletion of enterococci by antibiotics led to the upregulation of epithelial survival and barrier-protective genes, including serpins and other anti-apoptotic regulators. Conversely, mice harboring enterococci showed gene signatures associated with apoptosis and epithelial dysfunction. This was further supported by immunohistochemical evidence of increased apoptotic activity in these mice.

In mice treated with the triple antibiotic regimen, enterococci were depleted entirely, while only a limited number of residual bacterial genera were recovered from the mouse oral mucosa. Although we cannot conclusively rule out that the residual antibiotic-resistant bacteria were responsible for some of the host transcriptome changes, oral bacterial counts with this antibiotic regimen in the 5-FU OPC model are low to undetectable (7). Furthermore, in this study, we found mouse-to-mouse variability in the oral bacterial composition in the antibiotics group that cannot explain the significant barrier-compromising host response modification supported by our rigorous statistical framework. Therefore, the differential expression of genes related to mucosal barrier function was most likely caused by enterococci. Among these genes, three variants of the mouse serpina3 gene (n, k, and c) encoding Serprina3 were significantly overexpressed at the transcriptional level in mice treated with antibiotics. Serpins play important roles in host defense against pathogens, including inhibition of pathogen-derived proteases, binding of pathogens to cells, and enhancement of host immune responses (29). Serpina3n, which was significantly elevated at both mRNA and protein levels in mice depleted of enterococci, is an acute-phase protein synthesized in response to inflammation and tissue damage (30, 31) and is associated with epithelial anti-apoptotic function (32, 33). Seprina3 can act in an autocrine or paracrine fashion by entering cells and directly inhibiting intracellular caspase-3 activation (34). In addition to Serpina3n, the differential expression of Serpina3k and Serpina3c, which play roles in the protection of cells from oxidative stress and cell death (35, 36), was more highly expressed in antibiotics-treated mice, further supporting the notion that enterococci interfere with natural antioxidant host defense mechanisms that protect the mucosal barrier.

As a follow-up on these observations, we conducted proof-of-concept *in vitro* experiments using a murine enterococcal isolate to directly test the hypothesis that resident enterococci can induce oral epithelial apoptosis and/or necrosis. We found that *E. faecalis* strain Ef13 induced oral epithelial cell apoptosis *in vitro*, in line with previous observations of the pro-apoptotic effects of *E. faecalis* in osteoblasts (37). In addition to causing apoptosis, *E. faecalis* infection caused epithelial cell necrosis, which was significantly increased in cells co-infected with *C. albicans*, highlighting the potential for pathogenic synergy between the two organisms.

A major mediator of bacterially induced apoptosis in host cells is oxidative stress, principally due to H_2_O_2_ produced as a bacterial metabolic byproduct (38). We found that *E. faecalis* strain Ef13 produces H_2_O_2_, with both its magnitude and duration increasing during coincubation with *C. albicans. C. albicans* is protected from *E. faecalis*-induced oxidative damage by expressing catalase since both isogenic catalase mutants were rescued by adding catalase during co-culture, a viable alternative to gene complementation. Despite the increase in enterococcal H_2_O_2_ production, we did not find an increase in epithelial cell apoptosis above *E. faecalis* mono-infection with both organisms *in vitro*, possibly because under these epithelial coculture conditions, there is insufficient increase in H_2_O_2_ to cause a detectable increase in apoptosis; alternatively, epithelial cells may mount a greater protective antioxidant response when *C. albicans* is present.

Enterococcal extracellular release of H_2_O_2_ is dependent on growth conditions and available carbon sources (39, 40). When growing aerobically on glycerol as a carbon source, oxidation of glycerol by glycerol-3-P oxidase generates high amounts of H_2_O_2_ (40). Glycerol biosynthetic genes are strongly induced in *C. albicans* growing in the biofilm state, with glycerol accumulating within the biofilm structure. Moreover, it has been hypothesized that glycerol accumulation in mucosal biofilms may serve as a “stepping stone toward surface invasion” (41). It is thus possible that glycerol-based metabolic interactions both promote fungal-enterococcal mucosal biofilm formation and provide an alternative carbon source to *E. faecalis* co-colonizing these biofilms *in vivo*, promoting H_2_O_2_ production.

Although H_2_O_2_ was shown to play a role in *E. faecalis*-induced apoptosis, catalase only partially inhibited the significantly increased epithelial cell necrosis induced by both organisms together. Thus, we cannot rule out that an enterococcal pore-forming cytolysin, responsible for direct host cell damage (42), is also involved in synergistic cell death. Increased pore-forming toxin production by *S. aureus* is stimulated by *C. albicans* (43), and a similar mechanism of synergy may be involved with *E. faecalis*. Alternatively, *E. faecalis* may increase fungal epithelial toxicity by promoting hyphal morphogenesis and associated virulence gene expression in *C. albicans* (44).

One of the significantly under-expressed genes in antibiotics-treated mice was the gene encoding the calpain-1 protein, suggesting that enterococci induce higher expression levels of this protein. In prior work, we showed that *C. albicans* and *Streptococcus oralis* decreased epithelial E-cadherin levels by synergistically increasing μ-calpain, a proteolytic enzyme that facilitates fungal invasion (16). In the murine GI tract, *E. faecalis* is involved in regulating calpain-1 activity in response to inflammatory stimuli (45). Thus, it is possible that *E. faecalis* promotes oral mucosa barrier breach by synergizing with *C. albicans* to activate host enzymes such as calpain-1 that cleave epithelial junction proteins.

*E. faecalis* strain Ef13 and other murine enterococcal isolates express a gelatinase (GelE) that is involved in the reduction of intestinal barrier function by degrading E-cadherin (7, 46). However, this enterococcal enzyme may also have protective effects against *C. albicans*, since it is involved in cleaving the bacteriocin EntV into an active antifungal protein (47). Because of the significant *E. faecalis* clinical strain variation in the presence of the gene encoding gelatinase and its regulators (48), and the pH-dependent activity of the anti-fungal peptide derivatives of Entv (49), it is difficult to predict the role of these enterococcal proteins *in vivo*.

Transcriptomic analyses revealed that *C. albicans* mounts a mild oxidative stress response to *E. faecalis* (50), consistent with our finding that endogenous catalase is sufficient to protect fungal cells from low-level oxidative damage. Given the well-documented genome plasticity of enterococci (51), a limitation of our study is the use of a single murine *E. faecalis* strain. Strain-dependent variation in interactions with *C. albicans* has been observed *in vitro* (50). However, the majority of *E. faecalis* strains are potent producers of H_2_O_2_, and previous studies have shown that their impact on host cell apoptosis is strain independent and is reliant on bacterial metabolic activity (52). Thus, despite the use of a single murine enterococcal isolate, our findings are likely generalizable across multiple *E. faecalis* strains.

In summary, using complementary *in vivo* and *in vitro* approaches, our study supports a model in which dysbiotic expansion of *E. faecalis* under immunosuppressive conditions promotes fungal virulence through both oxidative and non-oxidative epithelial injury. *C. albicans* can amplify this effect by enhancing bacterial H₂O₂ production, establishing a feed-forward loop that may exacerbate mucosal damage *in vivo*. These findings highlight the need to consider bacterial-fungal interactions in managing OPC and suggest strategies aimed at modulating microbial community composition may serve as a promising adjunctive strategy in reducing disease severity in immunocompromised patients.

## Data Availability

Sequences were deposited at the NCBI GEO database under accession number GSE297794 at the following link: https://www.ncbi.nlm.nih.gov/geo/query/acc.cgi?acc=GSE297794 Microbial DNA sequencing raw data are accessible at the following link: http://www.ncbi.nlm.nih.gov/bioproject/531284.

## SUPPLEMENTAL MATERIAL

Fig. S1 msphere.00822-25-s0001.tif MA plot.

Fig. S2 msphere.00822-25-s0002.tif Functional classification of DEGs.

Fig. S3 msphere.00822-25-s0003.tif TUNEL staining.

Fig. S4 msphere.00822-25-s0004.tif Hydrogen peroxide production on MRS agar.

Fig. S5 msphere.00822-25-s0005.tif Effect of catalase on *Candida* oxidative damage

Fig. S6 msphere.00822-25-s0006.tif Epithelial cell apoptosis FACS assay.

Legends msphere.00822-25-s0007.docx Supplemental material legends

Supplemental data msphere.00822-25-s0008.xlsx DEG list.

Table S1 msphere.00822-25-s0009.docx Primer sequences.

ASM does not own the copyrights to Supplemental Material that may be linked to, or accessed through, an article. The authors have granted ASM a non-exclusive, world-wide license to publish the Supplemental Material files. Please contact the corresponding author directly for reuse.
