# Supplementary figures and images for: Enterococcus faecalis induces H₂O₂-mediated epithelial cell death and enhances Candida albicans virulence in oropharyngeal candidiasis
Source: mSphere. 2025 Dec 31;11(1):e00822-25. doi: 10.1128/msphere.00822-25 (PMC12838318; doi:10.1128/msphere.00822-25)

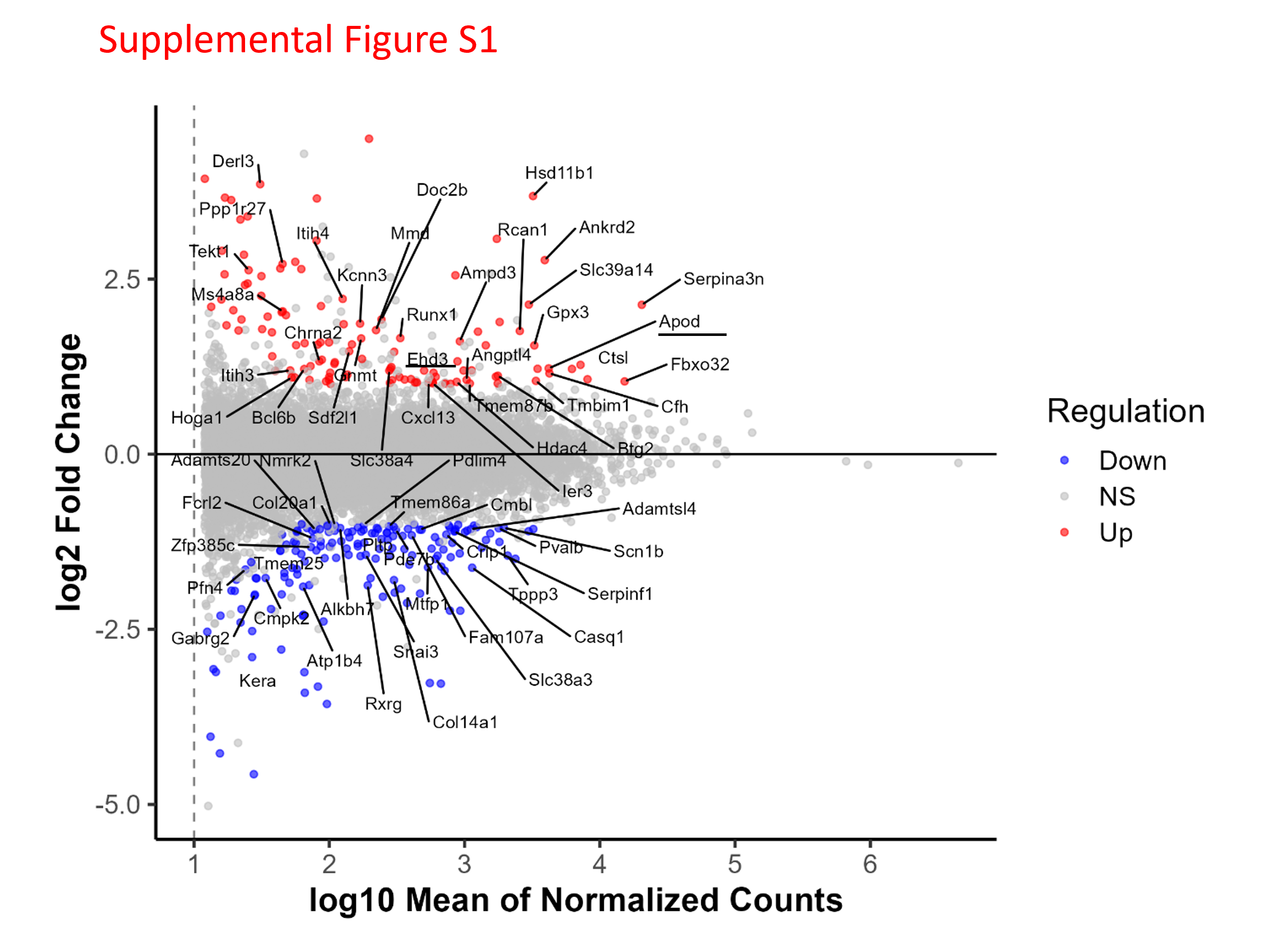

Supplement: Fig. S1 — MA plot. [file msphere.00822-25-s0001.tif]

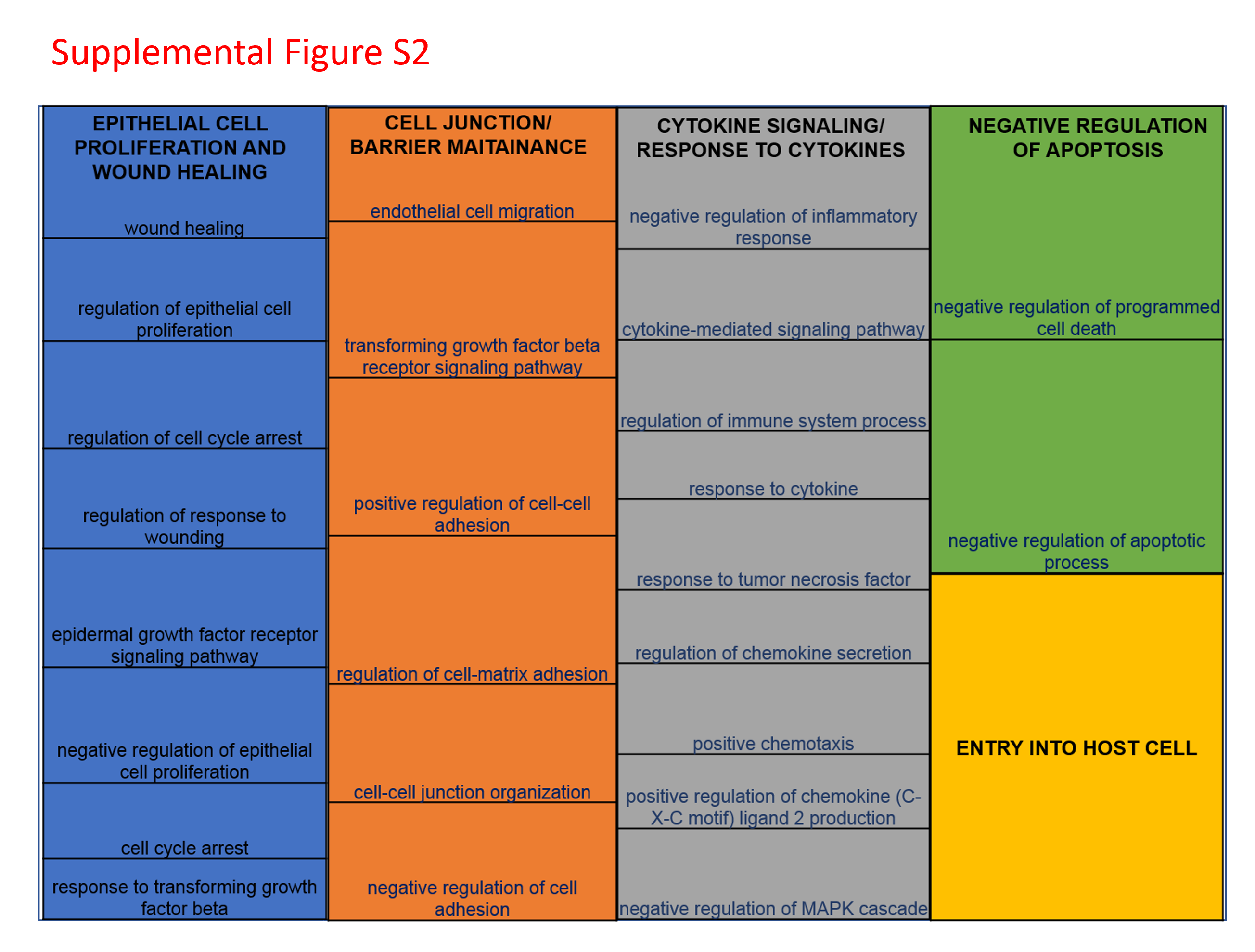

Supplement: Fig. S2 — Functional classification of DEGs. [file msphere.00822-25-s0002.tif]

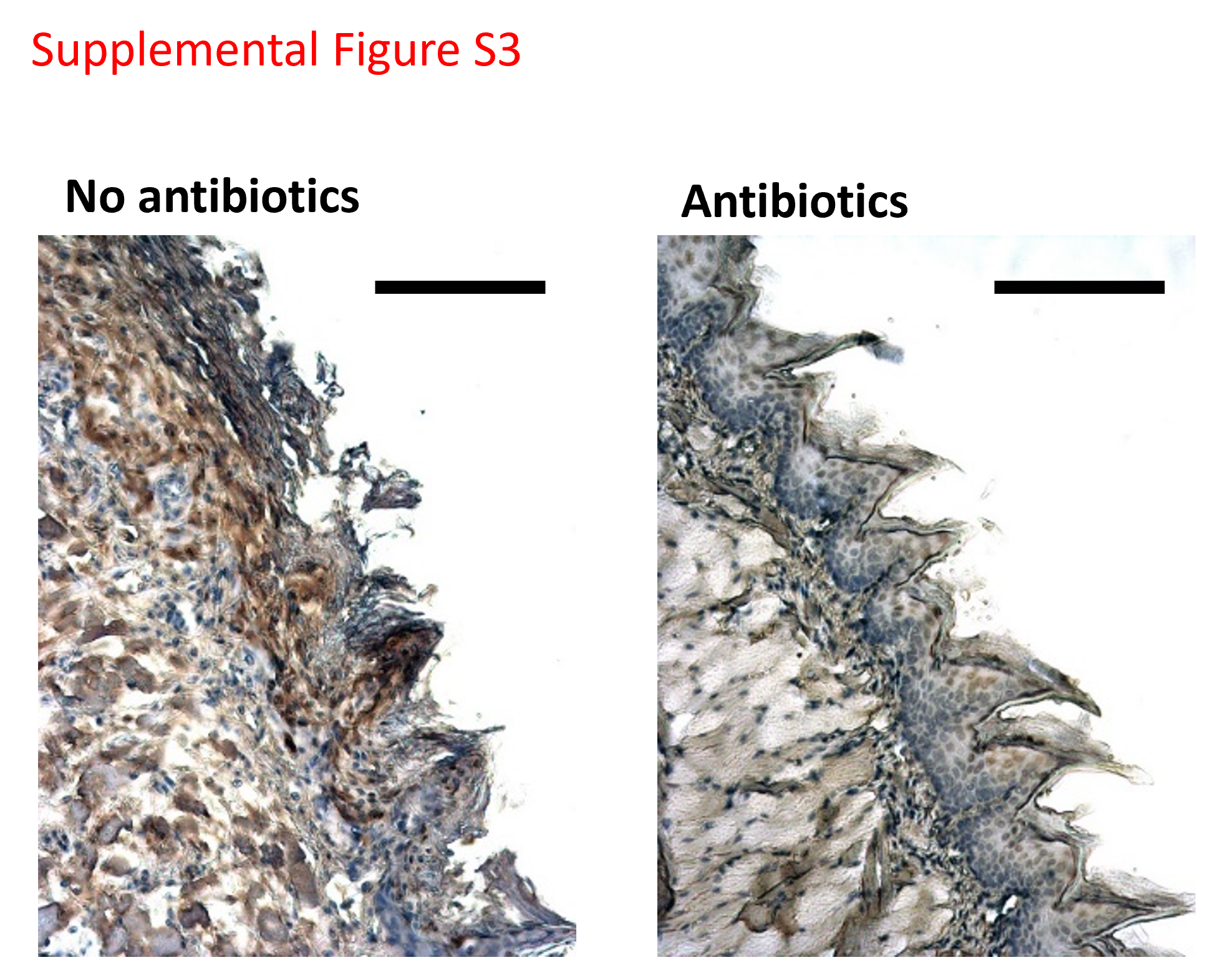

Supplement: Fig. S3 — TUNEL staining. [file msphere.00822-25-s0003.tif]

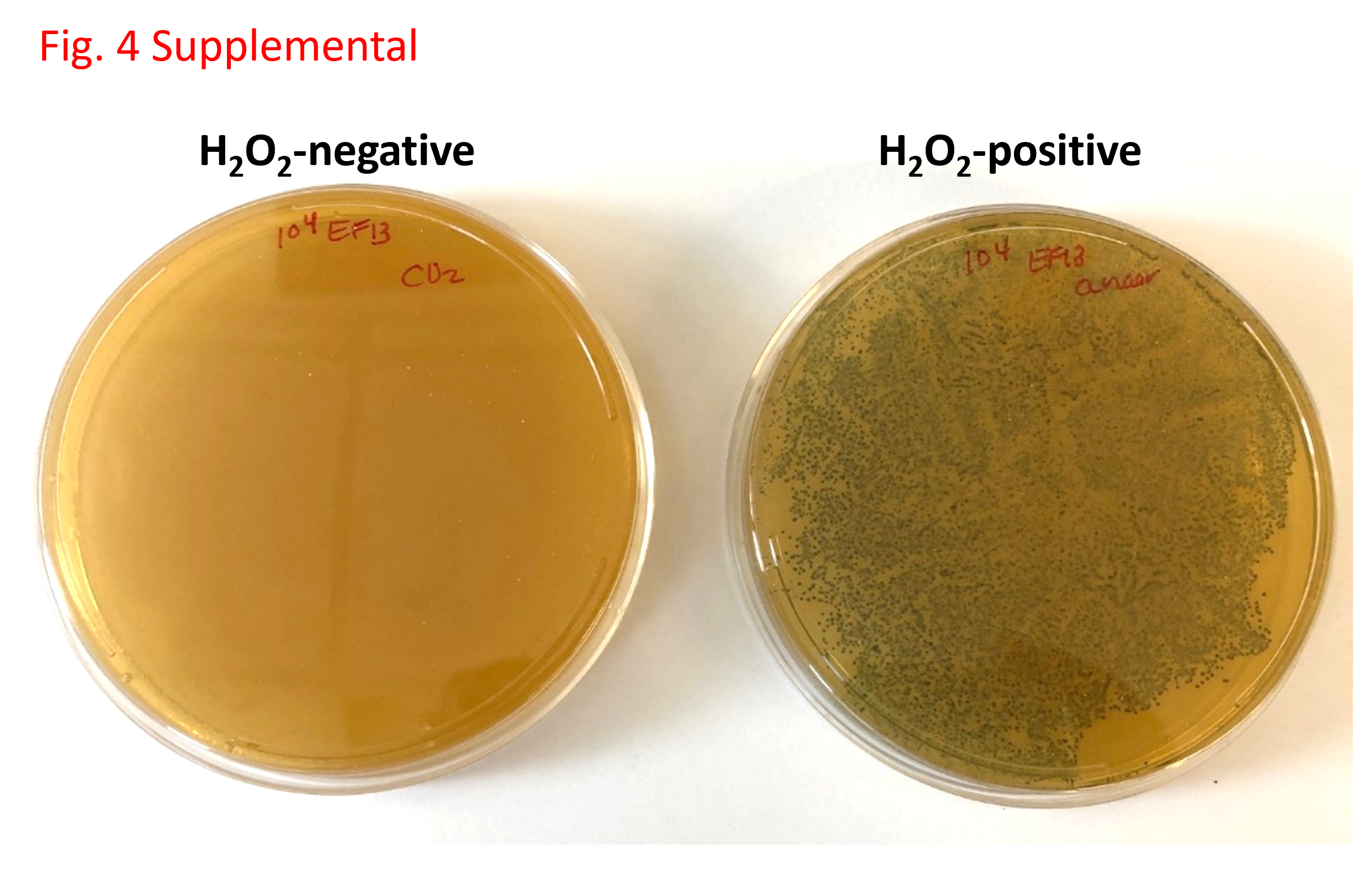

Supplement: Fig. S4 — Hydrogen peroxide production on MRS agar. [file msphere.00822-25-s0004.tif]

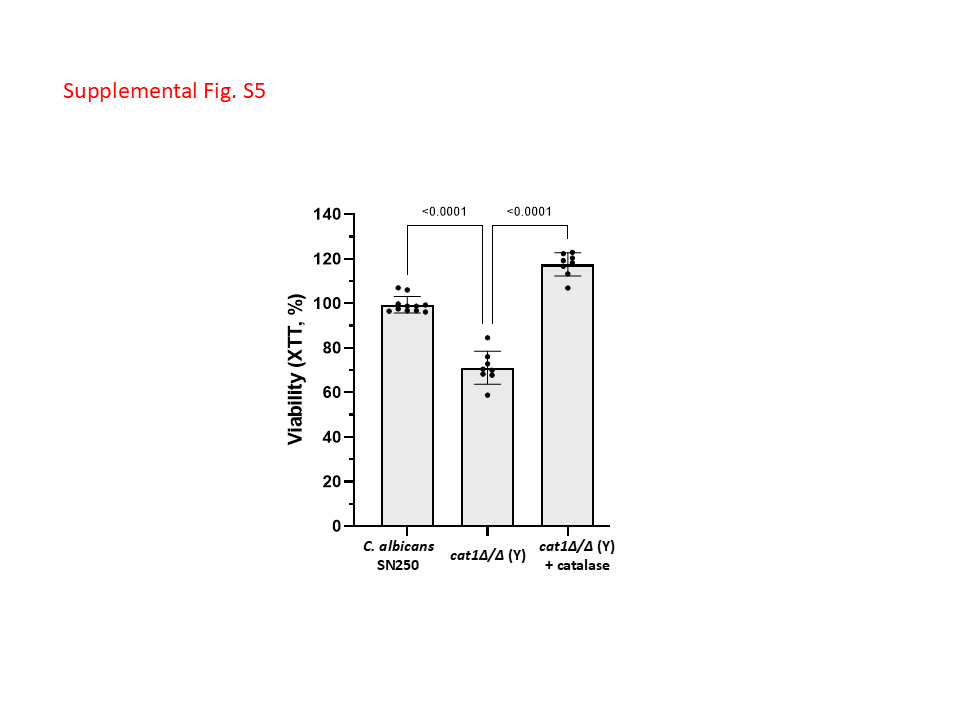

Supplement: Fig. S5 — Effect of catalase on Candida oxidative damage [file msphere.00822-25-s0005.tif]

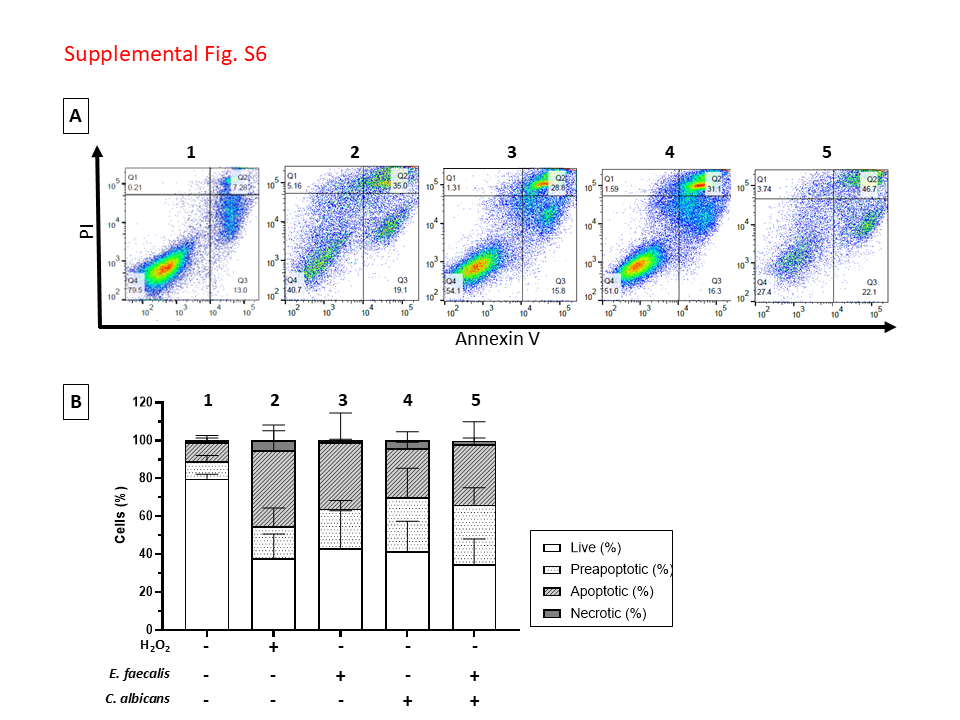

Supplement: Fig. S6 — Epithelial cell apoptosis FACS assay. [file msphere.00822-25-s0006.tif]
