# Supplementary material for: Enterococcus faecalis induces H₂O₂-mediated epithelial cell death and enhances Candida albicans virulence in oropharyngeal candidiasis
Source: mSphere. 2025 Dec 31;11(1):e00822-25. doi: 10.1128/msphere.00822-25 (PMC12838318; doi:10.1128/msphere.00822-25)
Supplement: Legends — Supplemental material legends [file msphere.00822-25-s0007.docx]

**Supplemental Figures**

**Figure 1 Supplemental**: **MA plot showing transcriptomic changes in antibiotic-treated versus untreated OPC mice**. The MA plot of normalized transcript counts in the *Candida*-infected antibiotic-treated group vs the group not treated with antibiotics supports the presence of broad transcriptional reprogramming following microbial depletion. Red dots represent significantly upregulated genes, blue dots indicate significantly downregulated genes, grey dots represent genes with no change (adjusted p < 0.05). Statistical analysis used DESeq2 with Benjamini–Hochberg correction.

**Figure 2 Supplemental:** Functional classification of differentially expressed genes (DEGs). Differentially expressed genes (|fold-change| ≥ 2, FDR ≤ 0.01) using GeneXplain and IPA Core Analysis were aggregated into five higher-order categories: Apoptosis, Epithelial proliferation/wound healing, Cytokine signaling/response, Cell junction/barrier maintenance, and Host-cell entry. The area of each rectangle is proportional to the number of DEGs in each subgroup.

**Figure 3 Supplemental:** **TUNEL staining of tongue tissues to assess apoptosis**. Representative tissue section stains are shown from the 5-FU-treated-*Candida*-infected (No antibiotics) and 5-FU-treated-*Candida*-infected Antibiotics groups. Bars=100 μm.

**Figure 4 Supplemental:** **H₂O₂ production by *E. faecalis* strain Ef13 under different oxygenation conditions.** Colonies of *E. faecalis* strain Ef13 growing on TMB-supplemented MRS agar. Under continuous aerobic growth conditions (left), this strain did not produce a pigment. After overnight anaerobic growth and subsequent exposure to air for 15 min (right), a blue pigment appeared in the colonies.

**Figure 5 Supplemental:** Effect of *C. albicans* catalase on oxidative damage from *E. faecalis*. *C. albicans* strain SN250 or an isogenic catalase homozygous deletion mutant (*cat1*Δ/Δ strain (Y)) were allowed to interact with *E. faecalis* for 2h at 1:10 fungal:bacterial cell ratio and fungal metabolic activity was measured by the XTT assay. Fungal metabolic activity was expressed as %fungal viability =x/y *100, where x is the OD450 of *C. albicans* with *E. faecalis* and y is the OD450 of *C. albicans* only. The metabolic activity of the catalase mutant is significantly compromised by *E. faecalis*, while the metabolic activity of the reference strain is not significantly affected. As with *cat1*Δ/Δ strain (X) (Figure 6B), added catalase (40U/well) rescued the mutant from oxidative damage. Results represent means ± SD from three independent experiments with technical triplicates. P-values shown are from Brown-Forsythe uncorrected One-Way ANOVA (Welch’s t test correction).

**Figure 6 Supplemental: Epithelial apoptosis following mono- and co-infection with *E. faecalis* and *C. albicans*.** OKF6/TERT-2 cells were infected with *E. faecalis*, *C. albicans* or their combination. Microbes were added on a transwell membrane (0.4 μm pore size) suspended over oral epithelial cells at 1 and 100 fungal and bacterial MOI, respectively and incubated for 5h. **A:** Gating after cells were stained with Annexin V and propidium iodide (PI) and sorted with FACS. Panels shown are from a representative of two independent experiments. **B:** Results are expressed as percentage of live, pre-apoptotic (Annexin V+), apoptotic (Annexin V+ PI+), or necrotic cells (PI+) over total cell numbers. Means ± SD are shown of results in two experiments with technical replicates*. E. faecalis*, *C. albicans* and their combination significantly increased pre-apoptotic and apoptotic cells, compared to control (p<0.05, ANOVA with the Uncorrected Dunn’s test). The percentage of apoptotic and pre-apoptotic cells was higher when the two organisms were combined compared to each species alone, but this difference did not reach statistical significance.

**Supplemental Table 1:** List of primers used in this study

**Supplemental data:** Complete list of Differentially Expressed Genes
